# Total Thoracic Aortic “Pericardialization” for Multilevel Prosthetic Graft Infection

**DOI:** 10.1016/j.jaccas.2026.110322

**Published:** 2026-10-07

**Authors:** Miralem Pasic, Sanjay Chaubey, Kang An

**Affiliations:** Department of Thoracic & Cardiovascular Surgery, Cleveland Clinic, Cleveland, Ohio, USA

**Keywords:** aorta, dissection, elephant trunk, endocarditis, pericardial tube graft, thoracic, valve replacement

## Abstract

**Background:**

Prosthetic aortic infection involving composite roots and frozen elephant trunk components presents an extraordinary challenge with high mortality. We present a complex aortic reconstruction using custom bovine pericardial conduits.

**Case Summary:**

A 55-year-old man with a history of a redo Bentall procedure and frozen elephant trunk implantation presented with extensive pan-aortic prosthetic infection, systemic septic emboli, and acute intracranial hemorrhage. Surgery focused on radical debridement and complete extrication of all synthetic material. The aortic root, ascending aorta, arch, and supra-aortic branches were entirely reconstructed using 4 intraoperatively hand-fashioned bovine pericardial tube grafts, creating a classic elephant trunk assembly. The patient was extubated on the day of surgery, discharged on day 7 without neurologic deficits, and remained infection-free at 3 months.

**Discussion:**

Radical excision and total aortic reconstruction using hand-made bovine pericardial grafts provide a completely biological, infection-resistant alternative for treating these highly complex conditions.

## Visual Summary

**Figure:**
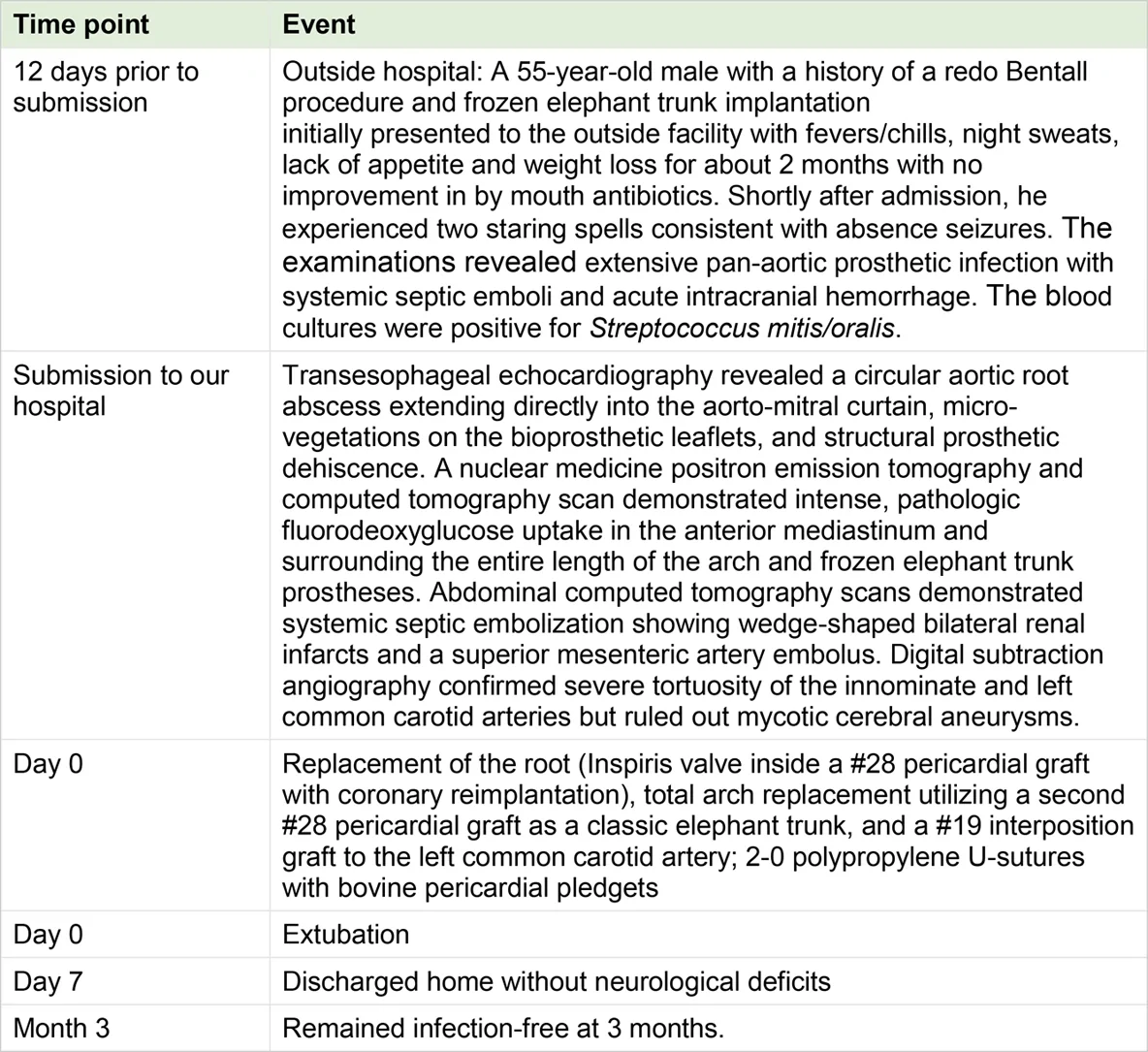

## History of Presentation

A 55-year-old man was transferred to our institution with a 2-month history of fevers, night sweats, fatigue, weight loss, and severe abdominal pain that had failed to respond to multiple courses of oral antibiotics. Blood cultures were positive for *Streptococcus mitis/oralis* following a recent dental cleaning performed without antibiotic prophylaxis. Shortly after admission, he experienced 2 blank staring episodes consistent with absence seizures. The preoperative course was complicated by a right cephalic vein deep vein thrombosis and a new 8-mm parenchymal intracranial hemorrhage identified after seizure-like activity. After neurosurgical consultation, heparin was held, and the patient was stabilized with levetiracetam.

## Past Medical History

The patient had a medical history of previous surgery for type A aortic dissection in 2012 followed by a complex second redo procedure in 2023 consisting of a redo Bentall composite graft (27-mm KONECT Bio-Conduit; Edwards) and a frozen elephant trunk arch replacement (28/30 × 100-mm Thoraflex Hybrid Branched Prosthesis; Terumo). Comorbidities included hypertension, chronic kidney disease stage 3a, obstructive sleep apnea, gastroesophageal reflux disease, and chronic fatigue syndrome. He also had a history of chronic proximal occlusion of the left subclavian artery manifesting as subclavian steal syndrome.

## Differential Diagnosis

The differential diagnosis for this presentation included:

1) Acute prosthetic valve endocarditis with root and arch graft involvement.
2) Periprosthetic mediastinal abscess or infected retrosternal hematoma.
3) Disseminated systemic malignancy or lymphoma.
4) Systemic vasculitis.

Given the patient’s surgical history, persistent bacteremia, and systemic inflammatory response, a catastrophic pan-aortic prosthetic graft infection was the primary diagnosis.

## Investigations

Laboratory findings demonstrated profound systemic inflammation: white blood cell count was 28.5 × 10^9^/L, C-reactive protein was elevated at 112.8 mg/L, and procalcitonin was 83.6 ng/mL.

Transesophageal echocardiography revealed a circular aortic root abscess extending directly into the aorto-mitral curtain, microvegetations on the bioprosthetic leaflets, and structural prosthetic dehiscence. A nuclear medicine positron emission tomography and computed tomography (CT) scan demonstrated intense, pathologic fluorodeoxyglucose uptake in the anterior mediastinum and surrounding the entire length of the arch and frozen elephant trunk prostheses, confirming prosthetic endocarditis (Figure 1). Abdominal CT scans demonstrated systemic septic embolization, showing wedge-shaped bilateral renal infarcts and a superior mesenteric artery embolus. Digital subtraction angiography confirmed severe tortuosity of the innominate and left common carotid arteries but ruled out mycotic cerebral aneurysms.

**Figure 1:**
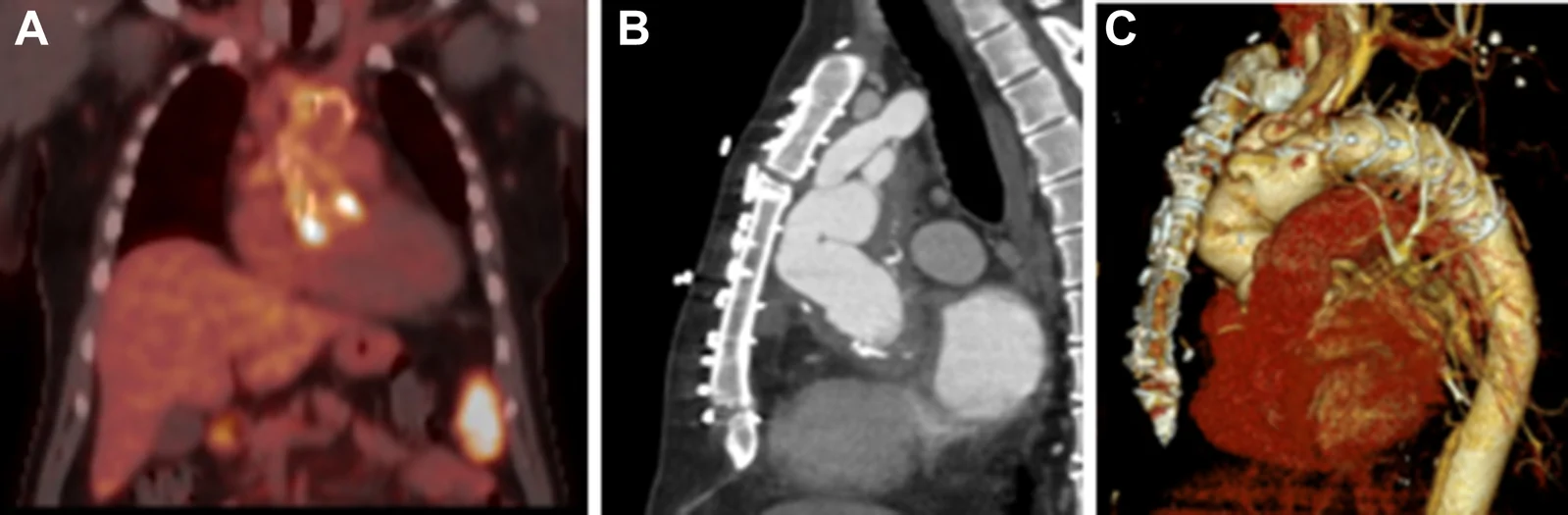
Preoperative Multimodal Imaging Confirming Pan-Graft Infection (A) Coronal positron emission tomography/computed tomography image demonstrating intense pathological radiotracer uptake and hypermetabolism surrounding the ascending aortic prosthesis, providing definitive diagnostic evidence of active prosthetic vascular graft infection. (B) Sagittal contrast-enhanced computed tomography angiography slice displaying the old prosthesis, kinked and completely adherent to the sternum with extensive, multiloculated retrosternal infected hematoma and periprosthetic abscess tracking directly behind the posterior sternal table. (C) Three-dimensional volume-rendered computed tomography reconstruction illustrating the structural complexity of the prior aortic arch repair, including the hybrid frozen elephant trunk stent-graft and supra-aortic debranching components, highlighting the high-risk anatomy ahead of the multiredo sternotomy.

## Management

Because of the extensive pan-aortic prosthetic infection, radical surgical intervention was planned. The patient was brought to the operating room. After induction of general endotracheal anesthesia and placement of appropriate lines, the patient was prepped and draped.

Before incision, 4 custom bovine pericardial tube grafts were hand-fashioned on the back table over #28 and #19 Hegar dilators. The longitudinal seams were constructed using a meticulous double-layer, continuous 5-0 polypropylene suture technique, featuring a primary geometric layer reinforced by an overlocked blanket stitch to ensure complete needle-hole hemostasis under arterial pressure. Surgical marker notations on the grafts (labeled S1, S2, and B) demonstrate intraoperative calibration to match the specific anatomical requirements for the aortic root, arch, and supra-aortic branches (Figure 2).

**Figure 2:**
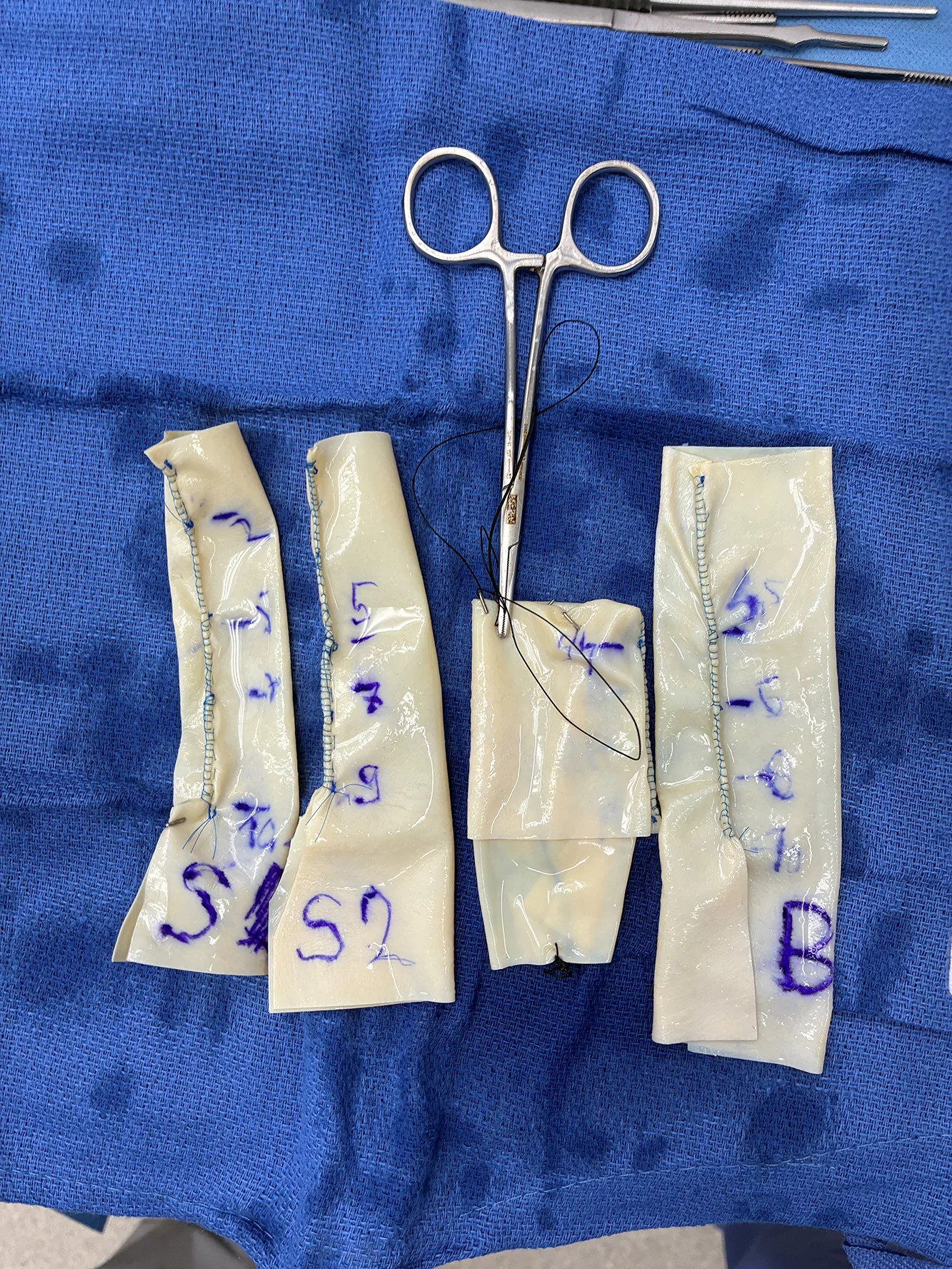
Intraoperatively Hand-Fashioned Bovine Pericardial Tube Grafts Back-table preparation of the total biological assembly. Custom geometric tube grafts have been hand-tailored from bovine pericardial patches. The precise, double-layer, continuous overlocked 5-0 polypropylene seams are clearly visible, calibrated specifically for the reconstruction of the aortic root/Bentall conduit, main aortic arch (classic elephant trunk), and the supra-aortic branches. Note the double-layer continuous overlocked suture technique used to ensure hemostatic integrity of the longitudinal seams. The hand-made bovine pericardial tubes were constructed with meticulous attention to hemostatic detail. The image highlights 3 points: 1) Suture technique: A double-layer, continuous suture line using 5-0 polypropylene. The primary layer provides the structural geometry of the tube, while the second layer is an overlocked (blanket) stitch visible along the longitudinal seam. This overlocking technique ensures that the edges of the pericardial sheet are securely compressed, preventing “fish-mouthing” and ensuring needle-hole hemostasis under arterial pressure. 2) Custom geometry: the completed grafts (labeled for specific anatomical sites) show tailored lengths and diameters with a perfectly linear suture line, ensuring a uniform caliber optimized for anatomical alignment. 3) Surgeon's markings: the custom surgical marker notations applied directly onto the grafts (eg, 2, 5, 7, and 10 cm) reflect the surgeon's precise intraoperative calibration performed on the back table to match the patient’s specific anatomical requirements for the aortic root, arch, and supra-aortic branches.

Preoperative CT analysis revealed that the left axillary artery was chronically occluded and the brachiocephalic prosthetic graft was severely stenosed and tortuous, precluding the use of the right axillary artery for cannulation. Consequently, safe arterial access was achieved via surgical cutdown and cannulation of the left femoral artery and vein. Because of the immediate retrosternal proximity of the infected synthetic material and the right ventricle (Figure 1), the third redo sternotomy was performed meticulously with an oscillating saw (Figure 3, Video 1). Cardiopulmonary bypass was held on standby during reentry to minimize the risk of premature catastrophic hemorrhage.

**Figure 3:**
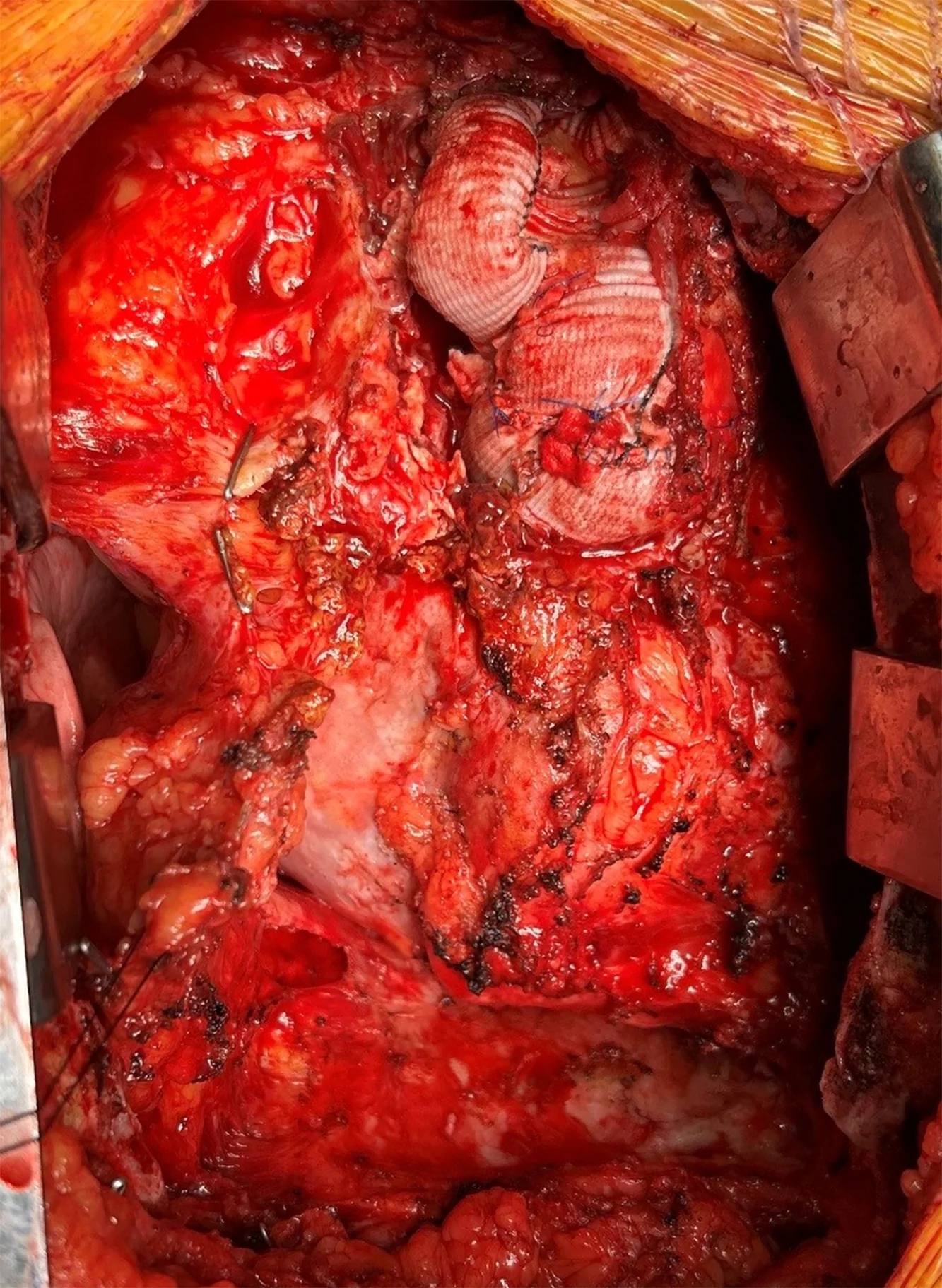
Wide-Angle View of the Multiredo Mediastinal Anatomy Comprehensive intraoperative orientation following rigid sternal re-entry using an oscillating saw. The image illustrates the complex anatomical landscape, intense fibrous adhesions, and the proximity of the infected composite graft to the posterior sternal table, requiring meticulous dissection under cardiopulmonary bypass standby. Orientation: the patient's head is at the top of the image (though not visible in the field), and the operating surgeon stands on the patient's right side. The figure corresponds to Video 1.

On entering the mediastinum, an extensive, firm inflammatory mass and a large periprosthetic abscess were encountered (Figure 4, Video 2). Bicaval venous cannulation was completed, the femoral venous cannula was retracted below the hepatic veins, and a retrograde cardioplegia cannula was placed directly into the coronary sinus. Cardiopulmonary bypass was initiated and systemic cooling begun. Retrograde Buckberg cardioplegia was used.

**Figure 4:**
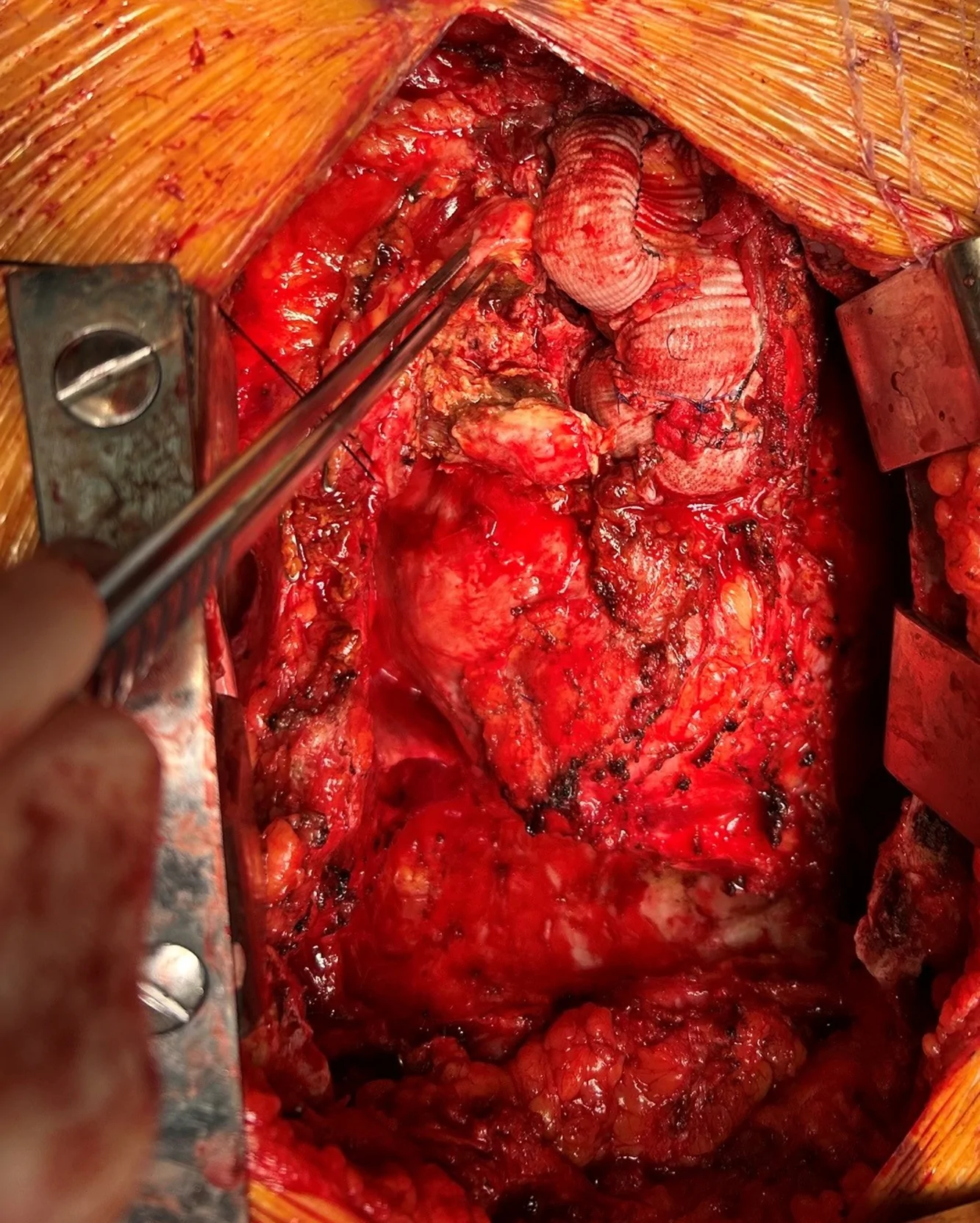
Gross Periprosthetic Abscess and Pan-Graft Infection Intraoperative view during the third redo sternotomy showcasing the massive infectious and inflammatory burden. The surgical forceps are pointing directly to a thick, purulent periprosthetic abscess wall and retrosternal infected hematoma surrounding the dehiscent, older synthetic aortic arch elements. Orientation: the patient's head is at the top of the image (though not visible in the field), and the operating surgeon stands on the patient's right side. The figure corresponds to Video 2.

The infected ascending aortic graft was clamped and incised, confirming macroscopic pan-prosthetic infection, a circumferential annular abscess, and degradation of the aorto-mitral curtain. Radical debridement of all synthetic material, infected thrombus, and necrotic tissue was performed. Intraoperative inspection after debridement showed no extensive mitral valve compromise and ruled out a Commando or hemi-Commando procedure. The aortic root was replaced by constructing a biological composite conduit using a #25 Edwards Inspiris bioprosthesis secured inside the first hand-made #28 bovine pericardial tube graft. Notably, 2-0 polypropylene mattress U-sutures reinforced with pericardial pledgets were used throughout the surgery instead of classical felt-pledgeted sutures. The coronary ostia were mobilized as buttons and directly reimplanted into this neoconduit (Bentall-DeBono configuration).

Systemic cooling was continued until a core temperature of 18 °C was reached, at which point circulatory arrest was initiated. Unilateral antegrade cerebral perfusion (total duration: 60 minutes) was delivered and alternated dynamically between the brachiocephalic trunk and the left common carotid artery. The infected frozen elephant trunk prosthesis was removed gently without aortic wall injury.

The second #28 hand-made pericardial graft was deployed into the descending aorta (zone 3) to function as a classic elephant trunk. The distal anastomosis was performed using continuous polypropylene sutures supported with additional polypropylene U-sutures. The arch component of the graft was then filled, clamped, and partial femoral perfusion was resumed at 2 L/min to limit visceral ischemia.

The brachiocephalic trunk was directly reimplanted into the neoarch pericardial graft. The left common carotid artery was reconstructed using a tapered #19 hand-made pericardial interposition graft made at the beginning of the surgery. The pericardial graft was interposed between the left common carotid artery and the pericardial arch graft. The chronically occluded left subclavian artery was left untouched. Full flow resumed, and the graft-to-graft anastomosis between the arch and the Bentall graft was completed during the rewarming (Figure 5, Video 3). The patient was successfully weaned from cardiopulmonary bypass under minimal inotropic support (Figure 5, Video 3). Instead of the planned transposition of the greater omentum into the pericardium, the entire pericardial assembly was covered with a 9 × 14 cm bovine pericardial patch before chest closure.

**Figure 5:**
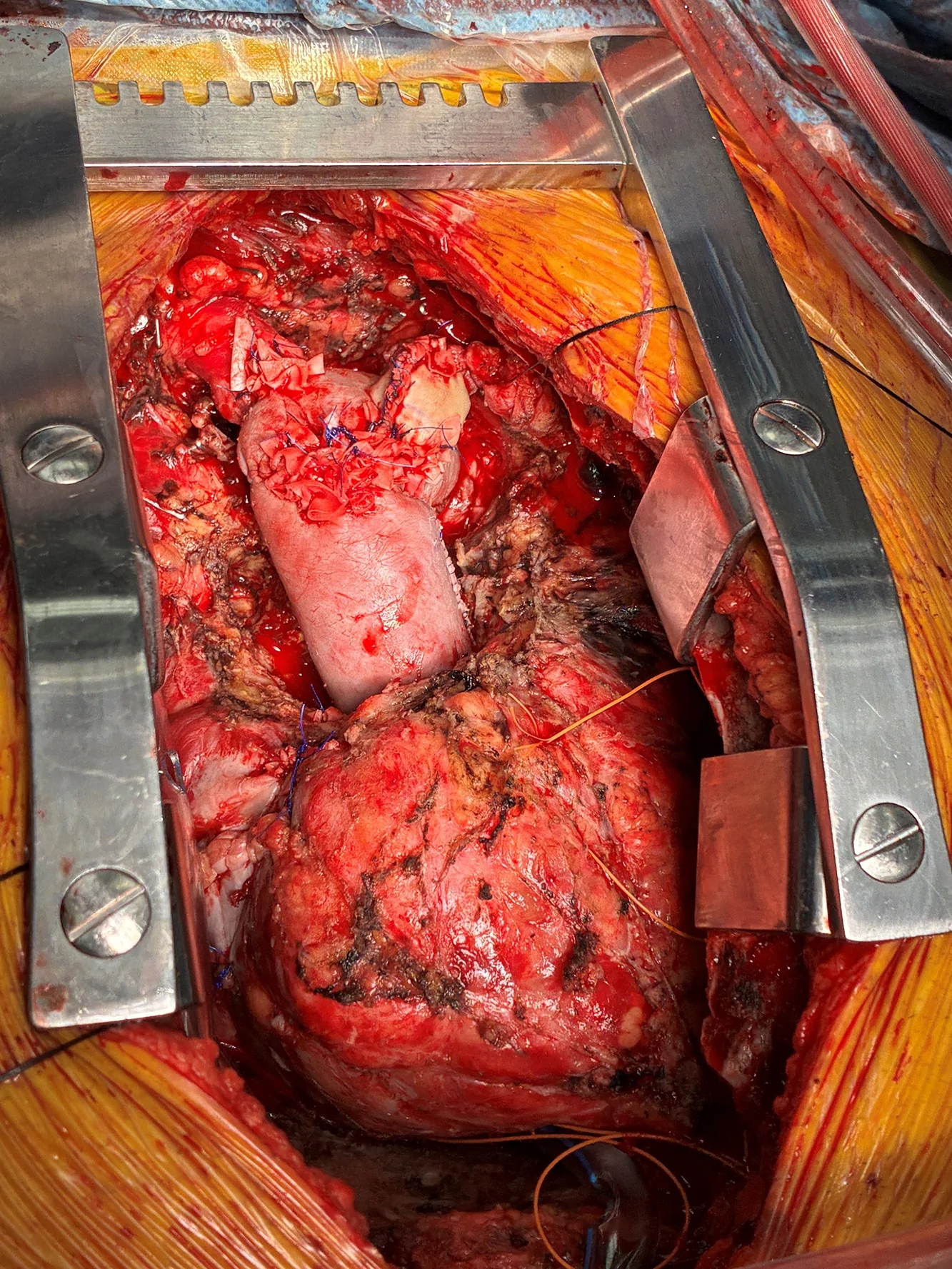
Final Root and Arch Reconstruction With Biological Material Final intraoperative view following successful total root, arch, and supra-aortic branch replacement using the completely biological, hand-fashioned bovine pericardial assembly. The image displays the completed distal and proximal anastomoses, demonstrating excellent structural integrity, complete clearance of the previous synthetic material, and full hemostasis before weaning from cardiopulmonary bypass. Orientation: the patient's head is at the top of the image (though not visible in the field), and the operating surgeon stands on the patient's right side. The figure corresponds to Video 3.

## Outcome and Follow-Up

The patient was extubated on the day of surgery. Notably, no neurologic complications occurred, which is particularly significant given the preoperative intracranial hemorrhage. He was discharged on postoperative day 7 to continue intravenous ceftriaxone for 6 weeks. Postoperative CT angiography confirmed a perfect anatomical result, demonstrating the patency of the pericardial composite graft, the arch reconstruction, and the elephant trunk. At the 3-month follow-up, the patient remains asymptomatic and has returned to normal daily activities. Stable imaging findings confirm the stability and patency of all pericardial grafts.

## Discussion

Multilevel prosthetic aortic endocarditis involving composite aortic root and frozen elephant trunk synthetic components represents a surgical milestone with high historical attrition rates. The traditional treatment paradigm relies on radical surgical debridement combined with synthetic graft or homograft replacement.^1^, ^2^, ^3^, ^4^ However, placing synthetic material back into a grossly infected mediastinal field heavily predisposes the patient to recurrent prosthetic valve endocarditis and catastrophic anastomotic dehiscence. Complete excision of all infected prosthetic material, radical local debridement, and the use of conventional homografts historically represent the gold standard for treating thoracic aortic graft infections.^2^ In line with this, current United States guidelines explicitly recommend the in situ placement of aortic allografts, deep veins, or silver-impregnated and rifampin-soaked prosthetic grafts for reconstruction within an infected field.^5^

Recent multicenter data^6^ and meta-analyses^7^^,^^8^ strongly endorse in situ reconstruction using biological or xenopericardial alternatives over the placement of synthetic materials within an infected mediastinal field to minimize reinfection rates. A total “pericardialization” of the thoracic aorta using hand-made bovine pericardial grafts presents a valuable alternative not only when conventional homografts are unavailable.^6^, ^7^, ^8^, ^9^, ^10^ It may also be used to avoid long-term complications in younger patients or for surgical technical reasons. First, a homograft is not an ideal landing zone for future endovascular interventions; because of its lack of predictable elasticity and potential for heavy calcification, it cannot provide an adequate anchoring zone. Attempting to place a stent in an allograft carries a high risk of endoleaks or even catastrophic graft rupture, whereas a hand-fashioned pericardial conduit may ensure a stable and secure platform for any necessary future stenting. Therefore, it is of paramount importance to not only replace the complete frozen elephant trunk prosthesis for infection control but also to prepare an adequate landing zone with a conventional elephant trunk.^10^ Second, in patients of a young age, severe calcification is frequently associated with homografts; such “porcelain” degeneration can render future reinterventions technically impossible. Only experienced redo-surgeons who have faced the nightmare of reoperating on a calcified allograft can fully appreciate this risk. Finally, the surgical handling and hemostatic properties of pericardium may be superior to the unpredictable quality of cryopreserved allografts, which can be thin-walled or brittle. Also, pericardial tissue possesses exceptional handling characteristics, supreme tensile strength, and natural resistance to bacterial colonization.^4^

## Conclusions

In cases of catastrophic, multilevel pan-aortic prosthetic infection, radical excision and total reconstruction using exclusively hand-made bovine pericardial grafts for the aortic root, arch, and elephant trunk offer a robust, infection-resistant biological alternative. This technique provides excellent surgical handling, immediate needle-hole hemostasis, and customized anatomical tailoring, while avoiding the long-term calcification associated with allografts. As noted by Czerny et al^9^ in their landmark paper published 15 years ago, this concept serves as an additional alternative to cryopreserved homografts that extends the armamentarium for treating patients with these highly complex conditions.

### Institutional Review Board Approval

Not required per institutional policy at the Cleveland Clinic for case reports/series involving patients.

**Take-Home Message**

• Hand-made bovine pericardial grafts can be used for the treatment of complex aortic graft infections.

## Funding Support and Author Disclosures

The other authors have reported that they have no relationships relevant to the contents of this paper to disclose.

## Appendix

Video 1 Radical Debridement and Tissue Clearance Intraoperative video sequence demonstrating the aggressive debridement of the anterior mediastinum and the tracking of chronic infection along the previous ascending aortic synthetic structures. The video highlights the extensive tissue mobilization required before safe cross-clamping and cardioplegia delivery could be established.

Video 2 Evacuation of Periprosthetic Abscess and Infected Hematoma Intraoperative video showing the direct exposure and surgical evacuation of a large, thick, purulent periprosthetic abscess and infected chronic hematoma directly abutting the previous synthetic arch and frozen elephant trunk components.

Video 3 Assessment of the Completed Hand-Fashioned Bovine Pericardial Assembly Final intraoperative video sequence demonstrating the completely reconstructed aorta in vivo after weaning from cardiopulmonary bypass. The camera close-up confirms a fully pulsatile, hemostatically stable bovine pericardial root-and-arch reconstruction, showcasing the fine suture lines of the tailored supra-aortic branches and the complete eradication of all synthetic material.
